# Effectiveness of Sensors Contact Metallization (Ti, Au, and Ru) and Biofunctionalization for *Escherichia coli* Detection

**DOI:** 10.3390/s18092912

**Published:** 2018-09-02

**Authors:** Sabina Górska, Artur Rydosz, Ewa Brzozowska, Marek Drab, Krzysztof Wincza, Andrzej Gamian, Sławomir Gruszczyński

**Affiliations:** 1 Department of Immunology of Infectious Diseases, Hirszfeld Institute of Immunology and Experimental Therapy, Polish Academy of Sciences, Weigla 12, 53-114 Wroclaw, Poland; sabina.gorska@iitd.pan.wroc.pl (S.G.); ezuziak@iitd.pan.wroc.pl (E.B.); marek.drab@iitd.pan.wroc.pl (M.D.); gamian@iitd.pan.wroc.pl (A.G.); 2 Department of Electronics, AGH University of Science and Technology, Al. Mickiewicza 30, 30-059 Krakow, Poland; artur.rydosz@agh.edu.pl (A.R.); krzysztof.wincza@agh.edu.pl (K.W.); 3 USI, Unit of Nano-Structural Bio-Interactions, Hirszfeld Institute of Immunology and Experimental Therapy, Polish Academy of Sciences, Weigla 12, 53-114 Wroclaw, Poland

**Keywords:** label-free biosensor, *E. coli* detection, microwave-based biosensor, ruthenium, titanium, gold, metallization

## Abstract

In designing a bacteria biosensor, various issues must be addressed: the specificity of bacteria recognition, the immobilization of biomolecules that act as the bacteria receptor, and the selectivity of sensor surface. The aim of this paper was to examine how the biofunctionalized surface of Ti, Au, and Ru metals reacts in contact with strains of *Escherichia coli* (*E*. *coli*)*.* The focus on metal surfaces results from their future use as electrodes in high frequency biosensors, e.g., resonant circuits or transmission-line sections. First, the surfaces of different metals were chemically functionalized with 3-aminopropyltriethoxysilane (APTES) and glutaraldehyde or with 3-glycidylooxypropyltrimethoxysilane (GPTMS) followed by *N*-(5-amino-1-carboxypentyl) iminodiacetic acid (AB-NTA) and NiCl_2_. Secondly, the lipopolysaccharide binding protein (LBP), polyclonal anti-*Escherichia coli* antibody and bacteriophage protein gp37 were tested as bacteria receptors. The selectivity and specificity have been confirmed by the Enzyme-Linked Immunosorbent Assay (ELISA) and visualized by scanning electron microscopy at low landing energies. We noticed that LBP, polyclonal antibody, and gp37 were successfully immobilized on all studied metals and recognized the *E*. *coli* bacteria selectively. However, for the antibody, the highest reactivity was observed when Ti surface was modified, whereas the bacteria binding was comparable between LBP and gp37 on the functionalized Ru surfaces, independent from modification. Thus, all surfaces were biocompatible within the scope of biosensor functionality, with titanium functionalization showing the best performance.

## 1. Introduction

**Figure 1:**
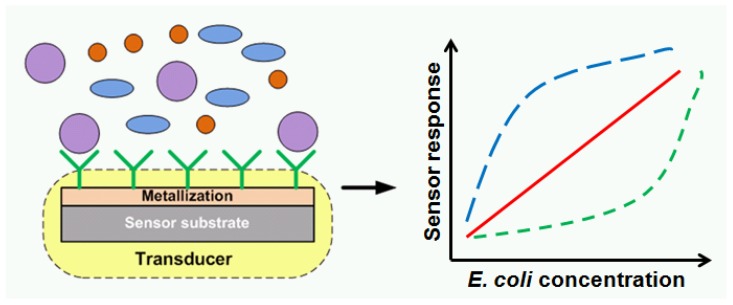
Schematic image of biosensor’s mechanism.

The rapid and precise detection of microorganisms through the development of modern biosensors accelerates implementation of appropriate treatment of infections and thus prevents major complications. Detection and identification of pathogens is based on conventional microbiological techniques, biochemical, molecular biology, or serological methods [1,2]. Even though Polymerase Chain Reaction (PCR) techniques such as: RT-PCR, qPCR, and Multiplex TaqMan PCR allow for fairly rapid identification, they show a number of disadvantages, such as: multi-step processes of labeling of different cell compartments and destructive preparation workflows [3]. To overcome these limitations label-free biosensing detection methods have been proposed [4]. The increasing number of publications on label-free methods confirms the possibility to utilize cost-effective biosensors for various cells detection [5,6,7,8]. Generally, a biosensor is constituted by a reactive layer able to recognize a specific biomolecule, and a transducer transforming the biochemical input interaction in a physical output signal as was shown in Figure 1. 

For optimal functioning of both parts of the biosensors, the reactive layer and the transducer, various issues must be addressed in designing a biosensor: the specificity of bacteria recognition, the immobilization of biomolecules that act as the bacteria receptor, and the sensitivity of sensor surface. This can lead to the advances in the development of rapid and highly sensitive biosensors. The biosensor surface mostly is a metal-based substrate (metal, metal oxide, or metal nitride) [9,10,11], however to work efficiently, the surface must be properly functionalized. Various surface modification strategies are available for bacteria sensors, among them the formation of self-assembling monolayers (SAMs) of molecules with suitable reactive termini is the most common procedure [12]. Covalent immobilization method using self-assembled layers of the biosensor surface is a promising technique and it has many advantages compared to others such as physisorption (physical adsorption) and bioaffinity immobilization [13]. If an optimal SAM is formed on the metal surface of the reactive layer, the maximum ratio between the number of bound molecules and the superficial area is reached, and the coupled molecules are properly oriented in the biological medium to maximize the binding events with the analyte. The chances of forming an efficient SAM are dependent both on the surface functionalization technique, but also on the method of preparation of the substrate: as the preparation method can influence the roughness and crystallinity of the metal surface [14]. Based on the literature review [15,16,17], it has been observed that in the formation of SAMs the step of immobilization of proteins is well established, however, optimal metal selection for surface metallization of substrate, most suitable for bacteria receptors, has not been yet sufficiently characterized. 

**Figure 2:**
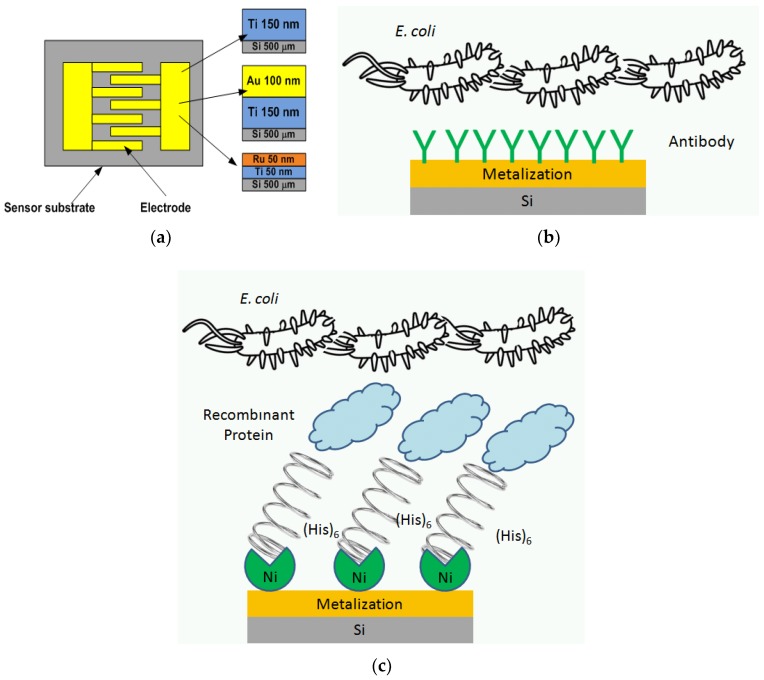
(**a**) Schematic image of the proposed sensors with investigated Ti, Au, Ru metallizations. Schematics of the biofunctionalization procedures: (**b**) using 3-aminopropyltriethoxysilane (APTES) and glutaraldehyde followed by polyclonal anti-*Escherichia coli* antibody and (**c**) 3-glycidylooxypropyltrimethoxysilane (GPTMS) and *N*-(5-amino-1-carboxypentyl) iminodiacetic acid (AB-NTA) and NiCl_2_ followed by lipopolysaccharide binding protein (LBP) or bacteriophage protein gp37 as a bacteria receptor.

The focus on metal surfaces is especially important for the whole class of electrical sensors, where the metallization of various substrates is utilized to fabricate typically passive circuits that after biofunctionalization respond to the binding events of the specified bacteria. The change of electrical response results from the changes of electric permittivity in the proximity of the sensor electrodes thus influences the capacitances of the sensor circuit, as shown schematically in Figure 2a where an interdigital capacitor is presented. The common feature of metals Ti, Au, and Ru, selected for this study is their excellent electrical conductivity, which equals 2.38 × 10^6^ S, 4.10 × 10^7^ S, and 1.40 × 10^7^ S respectively, and that all the three metals could be successfully applied in a sensor design. When considering a high frequency or microwave biosensor the conductivity of the metals impacts the attenuation of a circuit and the above listed values of conductivities correspond to the attenuation of a microstrip line section equal 9.25 dB/m, 2.22 dB/m, and 6.03 dB/m, calculated at 1 GHz, assuming characteristic impedance of a line equal to 50 Ohms on a 0.5 mm thick silicon substrate. To summarize the choice of a metal type is not critical from the electrical point of view but has an impact on the performance of sensor when exposed to chemical surface functionalization and bacteria binding. Thus it is justified to compare several metallization substrates and choose the best composition for electrical biosensors i.e., metal type, chemical surface modification, and biofunctionalization.

In the present study the effectiveness of three different metal contacts to detect the *Escherichia coli* were measured, i.e., titanium (Ti), gold (Au), and ruthenium (Ru). Figure 2 shows the schematic overview of the experiment. The metals were deposited by magnetron sputtering technique and chemically modified using 3-aminopropyltriethoxysilane (APTES) and glutaraldehyde (Figure 2b) or with 3-glycidylooxypropyltrimethoxysilane (GPTMS) followed by *N*-(5-amino-1-carboxypentyl) iminodiacetic acid (AB-NTA) and NiCl_2_ (Figure 2c). Next, the polyclonal anti-*Escherichia coli* antibody (Figure 2b) and lipopolysaccharide binding protein (LBP) or bacteriophage protein gp37 (gp37) (Figure 2c) were tested as a bacteria receptor. The functionalized layers’ structure and binding activity were analyzed with advanced modes of coating-free low-voltage field-emission scanning electron microscopy (LV-FESEM), energy-dispersive spectroscopy (EDS), and by enzyme-linked immunosorbent assays (ELISA).

## 2. Materials and Methods

### 2.1. Fabrication Process

The test structures were fabricated on n-type Czochralski (Cz) silicon wafers with resistivity of 5–7 Ω·cm. We used double-side polished (DSP) single crystal silicon wafers with 100-mm diameter and (1 0 0) crystal orientation. First, the full RCA cleaning procedure was performed to remove organic and inorganic contamination. The thermal oxidation was carried out to grow a layer of 760 nm-thick silicon dioxide. This process was started by growing thin oxide layer at 1000 °C and completed by wet oxidation at 1050 °C. Then, metal layers (Ti-150 nm, Ti/Au-250 nm and Ti/Ru-100 nm) were deposited by magnetron sputtering on both sides of the wafers, using the same deposition conditions for the front and the backside. Finally, wafer was diced into 10 mm × 10 mm samples. 

### 2.2. Characterization 

The structural analysis of the films was carried out by XRD using Panalytical Empyrean X-raydiffractometer (Malvern Panalytical Ltd, Royston, UK) with Co Kα radiation in grazing incidence GID configuration of 1°, 5°, and 15°. The chemical composition of the films were studied with EDS, including the Octane Silicon Drift Detector (EDAX, Weiterstadt, Germany) with beam energy of 12 keV and a beam current of 4 nA. The thin films morphology was characterized by Scanning Electron Microscopy using FEI VERSA 3D dual-beam system (FEI, Eindhoven, The Netherlands). 

### 2.3. Bacteria Growth Condition

Six *Escherichia coli* (*E*. *coli*) strains: B (PCM1935), 056 (PCM 2372), K12 (PCM 2560), 0111 (PCM 418), 024 (PCM195), and 0104 (PCM 270) were cultivated in Luria-Bertani medium at 37 °C with shaking at 120–150 rpm until optical density (OD) of 600 nm reached value 1 (10^9^ CFU/mL), whereas *Lactobacillus rhamnosus* LOCK 0919 was cultivated in de Man, Rogosa, and Sharpe medium under microaerofilic conditions at 37 °C until OD of 600 nm reached value 1 (10^9^ CFU/mL).

### 2.4. Enzyme-Linked Immunosorbent Assay (ELISA)

#### 2.4.1 Whole-Bacterial Cell ELISA

Flat-bottomed 96-well MaxiSorp plates (Nunc, Roskilde, Denmark) were coated with 200 µL of whole bacteria (0.25 mg of lyophilized bacterial mass/1 mL of PBS, 100 (10^8^ CFU/mL), 10 (10^7^ CFU/mL), and 1 (10^6^ CFU/mL) µL of alive bacterial culture in PBS). Plates were centrifuged at 600× *g*, 4 °C for 20 min and the liquid was discarded carefully. Then, 250 µL of 0.1% glutaraldehyde was added to plate wells and incubated at room temperature (RT) for 30 min. The liquid was discarded carefully and plates washed 3 times for 15 min each wash step at RT with PBS-T (PBS + 0.05% Tween 20). Plates were blocked at RT for 2 h with 250 µL of 0.1% bovine serum albumin (BSA, Sigma Aldrich, St. Louis, MI, USA) in PBS with 0.1 M glycine (Sigma Aldrich). Plates were subjected to three washes with PBS-T buffer and then incubated overnight at 4 °C with 250 µL of 0.25% BSA. After washing three times with PBS-T, plates were incubated with 100 µL of anti-*Escherichia coli* antibody conjugated with horseradish peroxidase (HRP) (1:2000, Abcam, Cambridge, MA, USA) at 37 °C for 1 h. Finally, the plates were washed five times and the 3,3’,5,5’-tetramethylbenzidine (TMB) substrate was added to yield colored reaction. To stop the reaction 50 µL of 2 M H_2_SO_4_ was added and finally, the plates were read at 450 and 570 nm on a plate reader (Biotek, Winooski, VT, United States). Data are expressed as a mean and standard error of the mean (M ± SEM). Statistical analysis was performed by one-way ANOVA test (Tukey’s multiple comparisons test) using Prism 5.06 software (GraphPad, San Diego, CA, USA). *P* values of < 0.05 were considered significant.

#### 2.4.2. Protein ELISA

Flat-bottomed 96-well MaxiSorp plates (Nunc) were coated at 4 °C overnight with 200 µL of lipopolysaccharide binding protein (0.25 µg/well, LBP, Sigma Aldrich), adhesin gp37 solution (0.25 μg/well, prepared in our laboratory as mentioned in [18]), and anti-*Escherichia coli* antibody (1:200/well, Abcam) solution in 50 mM carbonate/bicarbonate buffer, pH 9.6. Plates were washed three times with wash buffer: PBS-T (phosphate buffer + 0.05% Tween 20) and blocked for 1 h at 25 °C with 1% BSA in PBS. Again, plates were washed 5 times with PBS-T and incubated with 100 µL of whole bacterial solution in PBS (0.25 mg of bacterial mass/1 mL of PBS; 100, 10, and 1 µL of alive bacterial culture) for 2 h at 25 °C. Following 3 washes, the rabbit polyclonal serum anti-*Escherichia coli* conjugated with horseradish peroxidase (HRP) were applied (1:2000, Abcam) and incubated at 37 °C for 1 h. Finally, the plates were washed five times and 3,3’,5,5’-tetramethylbenzidine (TMB) substrate was added to yield colored reaction. To stop the reaction 50 µL of 2 M H_2_SO_4_ was added, and finally, the plates were read at 450 and 570 nm on a plate reader (Biotek). Data are expressed as a means and standard errors of the means (SEM). Statistical analysis was performed by one-way ANOVA test (Tukey’s multiple comparisons test) using Prism 5.06 software (GraphPad, San Diego, CA, USA). *P* values of < 0.05 were considered significant.

### 2.5. Biofunctionalization

#### 2.5.1. Biofunctionalization by His-Tagged LBP Protein and Bacteriophage His-Tagged gp37 Adhesin

The biofunctionalization of the metal surface was performed according to the method previously described by us [19,20]. Briefly, the metal surfaces were immersed in 0.01% acetic acid mixed with 2% 3-glycidylooxypropyltrimethoxysilane (GPTMS) solution for 3 h at 90 °C, washing, and incubating for 16 h in 10 mM NaHCO_3_ mixed with 20 mM *N*-(5-amino-1-carboxypentyl) iminodiacetic acid (AB-NTA) at 20 °C. The surfaces were subsequently washed with PBS-T, immersed for 2 h at 60 °C in 10 mM NiCl_2_ with 5 mM glycine, washed with PBS-T, and then finally immersed in bacteriophage His-tagged gp37 adhesin (0.25 µg/well) or His-tagged LPB protein (0.25 µg/well) solution in PBS to provide the biomolecules immobilization. The surfaces with proteins were washed and immersed in *E*. *coli* B, *E*. *coli* 011, and *L*. *rhamnosus* LOCK 0919 solution for 2 h at room temperature. Following 3 washes, the surfaces were tested by scanning electron microscopy at low voltage (LV-SEM). For the quantitative control of biofunctionalization, after the surfaces were washed, the rabbit polyclonal serum anti-*Escherichia coli* conjugated with horseradish peroxidase (HRP) was applied (1:2000, Abcam) and incubated at 37 °C for 1 h. Samples were washed five times with PBS and finally, the 3,3’,5,5’-tetramethylbenzidine (TMB) substrate was added to yield colored reaction. To stop the reaction 2 M H_2_SO_4_ was added, and finally, the plates were read at 450 and 570 nm on a plate reader (Biotek). Data are expressed as a means and standard errors of the means (M ± SEM). Statistical analysis was performed by one-way ANOVA test (Tukey’s multiple comparisons test) using Prism 5.06 software (GraphPad, San Diego, CA, USA). *P* values of < 0.05 were considered significant.

#### 2.5.2. Biofunctionalization by Polyclonal Anti-*Escherichia coli* Antibody

The biofunctionalization of the metal surface was performed as follows. The metal surfaces were immersed in 5% 3-aminopropyltriethoxysilane solution (APTES) in methanol for 3 h at 25 °C, washed three times with methanol and distilled water, and incubated for 30 min in 5% glutaraldehyde solution at 25 °C. The surfaces were subsequently washed three times with PBS-T, immersed overnight at 4 °C in anti-*Escherichia coli* antibody (1:200/well, clone nr, Abcam) solution in PBS, washed five times and blocked for 1 h at 25 °C with 1% BSA in PBS. The surfaces coated with proteins were washed five times and immersed in *E*. *coli* B, *E*. *coli* 011, and *L*. *rhamnosus* LOCK 0919 solution for 2 h at room temperature. Following 3 washes, the surfaces were tested by field emission scanning electron microscopy, in a low voltage approach (LV-FESEM). For the quantitative control of biofunctionalization, after the surfaces were washed, the rabbit polyclonal serum anti-*Escherichia coli* conjugated with horseradish peroxidase (HRP) was applied (1:2000, Abcam) and incubated at 37 °C for 1 h. Samples were washed five times with PBS and finally, the 3,3’,5,5’-tetramethylbenzidine (TMB) substrate was added to yield colored reaction. To stop the reaction 2 M H_2_SO_4_ was added, and finally, the plates were read at 450 and 570 nm on a Plate reader (Biotek). Data are expressed as a means and standard errors of the means (M ± SEM). Statistical analysis was performed by one-way ANOVA test (Tukey’s multiple comparisons test) using Prism 5.06 software (GraphPad, San Diego, CA, USA). *P* values of < 0.05 were considered significant.

### 2.6. Low-Voltage Field-Emission Scanning Electro Microscopy LV-FESEM and Real-Time Energy Filtering Mapping

For imaging of mixed structures, involving biological object (bacteria) and nanostructure-patterned material (biosensor electrodes) in the same field of view, the electron microscopy (EM) provides particularly useful insights. In an adopted low-voltage (LV) mode (LV-FESEM), due to the low energies of the incident beam electrons, such as 800 eV used in this study, a sufficient image contrast can be generated directly from the endogenous components of a biological sample, without coating or contrasting, thus compatible with chemical mapping [21]. The coating-free approach within LV-FESEM, which omits extraction, allows for analysis of bacteria–biosensor interactions in two-complementary aspects: to image the topography (SE2 electrons detected with Everhart-Thornley (ET) detector) and to generate chemical contrasts (low-loss back-scattered electrons LL-BSE, detected with energy-selective back-scattered electrons (EsB) detector) [22]. The energy filtering of the back-scattered electrons, due to their single-inelastic interaction memory allows extraction of chemical contrast, capable to detect and distinguish thin layers of metal, silicon, organic coating, and fine structure of biological objects [21]. The energy filtering is based on the adaptation of retarding field analyzer (RFA) concept with the electrical grid potential set to 600 V. Under these conditions the EsB detector was reached by back-scattered electrons with low-loss energy 200 eV as a result of potential difference between 800 eV (incident beam) and 600 eV (retarding grid potential); a detailed description of the method was described previously [22]. The aspects of topography/texture and chemical contrast were spatially correlated at high resolution.

## 3. Results

### 3.1. XRD/EDS

**Figure 3:**
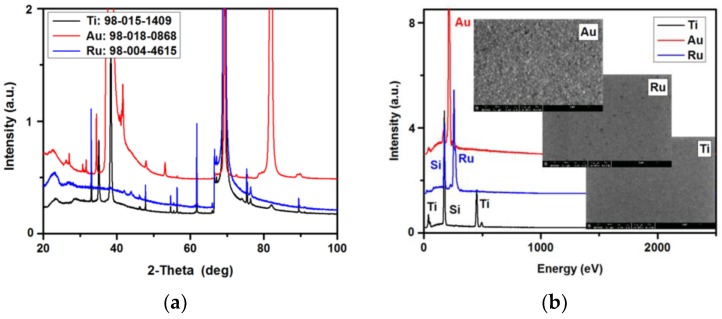
(**a**) XRD pattern of deposited metals, (**b**) EDS spectra of deposited metals with SEM images of the representative metals’ surfaces.

The XRD (Figure 3a) and EDS (Figure 3b) measurements have shown that pure metals (fully crystallized) were deposited without any impurities, however, the Ti and Ru samples were self-oxidized: 3.42 wt % and 0.77 wt % for Ti and Ru metal contacts, respectively.

### 3.2. Sensitivity of Polyclonal Anti-Escherichia coli Antibody Conjugated with Horseradish Peroxidase

**Figure 4:**
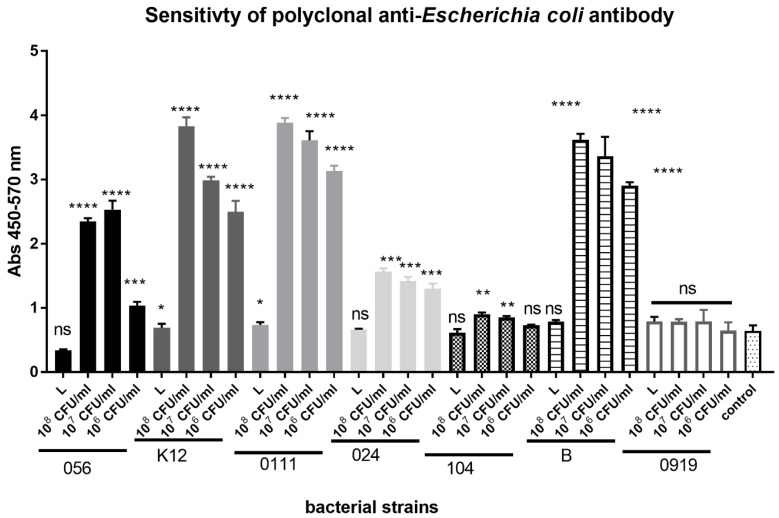
The reactivity in the ELISA test of polyclonal anti-*Escherichia coli* antibody conjugated with HRP (1:2000) with *Escherichia coli* strains: 056, K12, 0111, 024, 104, B, and *Lactobacillus rhamnosus* LOCK 0919 (919). L-0.25 mg of lyophilized bacterial mass/1 mL of PBS; 10^8^ CFU/mL, 10^7^ CFU/mL, 10^6^ CFU/mL of alive bacterial culture in PBS. Pooled results from three independent experiments are shown. *, *P* < 0.05; **, *P* < 0.01; ***, *P* < 0.001; ****, *P* < 0.0001; ns- non-significant.

The sensitivity of the polyclonal anti-*Escherichia coli* antibody was tested in whole-bacterial cell ELISA (Figure 4). We observed differences in anti-*Escherichia coli* antibody binding to six strains of *E. coli* that depended on bacterial strain, status of bacteria vitality, and concentration. Generally, we observed higher values of absorbance when the live bacteria were used compared to the lyophilized ones. The level of absorbance depended on bacteria concentration, however 10^6^ CFU/mL of bacteria was detected with high absorbance value. Interesting, binding of antibody to *E*. *coli* B, *E*. *coli* 0111, *E*. *coli* 056, and *E*. *coli* K12 was higher than to *E*. *coli* 024 and *E*. *coli* 104. The *Lactobacillus rhamnosus* LOCK 0919 was used as a negative control and the level of absorbance was comparable to the control (a well without bacteria). 

### 3.3. Specificity and Selectivity of LBP, gp37 Adhesin and Polyclonal Anti-E. coli Antibody

**Figure 5:**
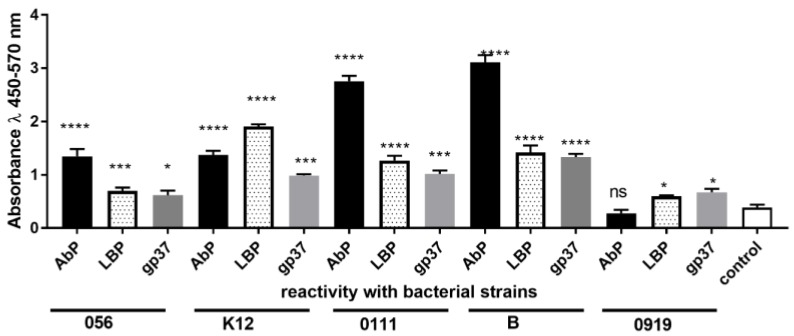
The reactivity in the ELISA test of lipopolysaccharide binding protein (LBP), adhesin gp37 (gp37), polyclonal anti-Escherichia coli antibody (AbP) with *Escherichia coli* strains: 056, K12, 0111, B, and *Lactobacillus rhamnosus* LOCK 0919 (919). Pooled results from three independent experiments are shown. *, *P* < 0.05; ***, *P* < 0.001; ****, *P* < 0.0001; ns., non-significant.

The specificity and selectivity of biologically active LBP, adhesins gp37 from T4 phage and polyclonal anti-*E*. *coli* antibody was evaluated and Figure 5 shows the results. Based on the sensitivity results of anti-*Escherichia coli* antibody conjugated with horseradish peroxidase, in the present experiment we used only four alive *E*. *coli* strains and *L*. *rhamnosus* LOCK 0919 as negative control. We observed that all biologically active bacteria were recognized by LBP, gp37 adhesin, and polyclonal anti-*E*. *coli* antibody, however the highest value were observed in reaction between alive bacteria and polyclonal antibody.

### 3.4. Evaluation of Escherichia coli Sensitivity and Specificity Detection on Various Protein-Modified Different Metal Surfaces

With the performed experiment, the effectiveness of different sensor surface (Ti, Au, and Ru) and biofunctionalization preparation to detect the bacteria were performed. Two separately different methods were used to confirm and visualize results: colorimetric reaction in ELISA and SEM imaging.

**Figure 6:**
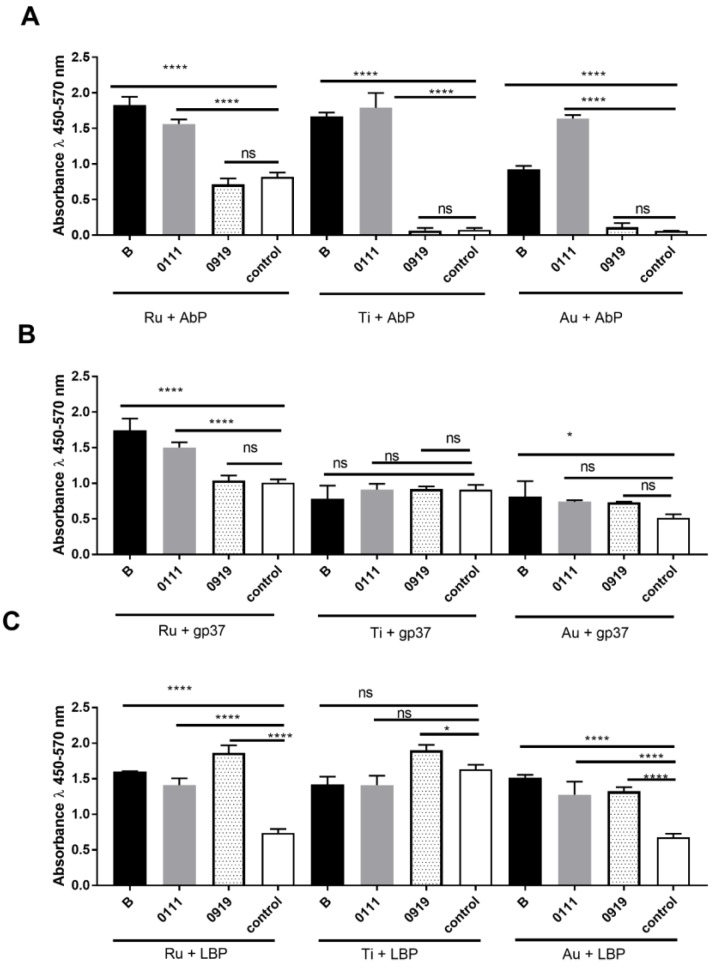
The reactivity of (**A**) polyclonal anti-*Escherichia coli* antibody, (**B**) adhesin gp37, (**C**) lipopolysaccharide binding protein (LBP) immobilized on different metal surface Ru, Ti, or Au by glutaraldehyde (**A**) or NiCl_2_ (**B**,**C**) with *Escherichia coli* strains: 056, K12, 0111, B, and *Lactobacillus rhamnosus* LOCK 0919 (0919). Pooled results from three independent experiments are shown. *, *P* < 0.05; ****, *P* < 0.0001; ns-non-significant.

Figure 6 shows the reactivity between: bacteria (*E*. *coli* and *L*. *rhamnousus*) and active biomolecules which selectively recognize the *E*. *coli* strains (AbP, gp37, and LBP) immobilized on different metal surfaces. For example, we observed that absorbance value of *E*. *coli* detection (both B and 0111 strain) is very high (varied from 1.0 to 2.0) when the AbP were immobilized on all metal surfaces, however the ratio between absorbance of *E*. *coli* and *L*. *rhamnosus* (0919, negative control) and/or control (only PBS) was the highest when Ti was functionalized. The absorbance ratio between *E*. *coli* B/*L*. *rhamnosus* detection by AbP immobilized on Ti is 1.8/0.1 = 18, whereas *E*. *coli* B/*L*. *rhamnosus* detection by AbP immobilized on Au is 1.0/0.15 = 6.67. Based on the absorbance values, or more precisely the ratios between positive control and negative control and/or positive control and control (PBS only), we concluded that the most sufficient *E*. *coli* B or 0111 strains’ detection is the AbP immobilization on Ti metal surface, or gp37 immobilization on Ru metal surface. 

For imaging of mixed structures, involving biological object (bacteria) and nanostructure-patterned material (biosensor electrodes) in the same field of view, the electron microscopy (EM) provides particularly useful insights. The coating-free approach within LV-FESEM mode, which omits extraction, allows for analysis of bacteria–biosensor interactions in two-complementary aspects: to image topography/texture and to extract chemical contrast, both aspects spatially correlated at high resolution [21]. 

**Figure 7:**
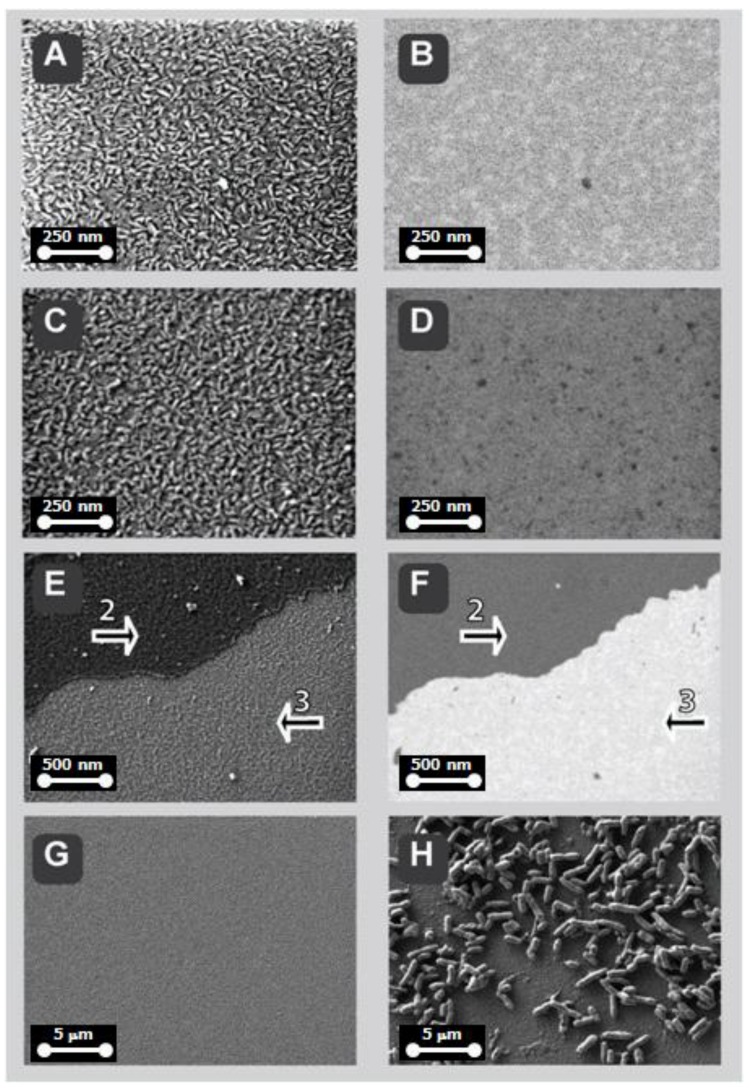
Biofunctionalization of biosensors for binding of bacteria *Escherichia coli* visualized by scanning electron microscopy, SEM, for titanium-metalized silicon chip. Bare metal (**A**,**B**), was coated with APTES (**C**,**D**), then coupled to the antibody (**E**,**F**) specific for *Escherichia coli* bacteria. The bacteria binding is shown in (**H**) as a result of specific interaction bacteria *Escherichia coli*/antibody anti-*Escherichia coli*, while the negative control (**G**) (non-matched *Lactobacillus rhamnosus* bacteria/antibody anti-*Escherichia coli*) showed no binding. The topography/texture imaging (ET detector) was (pixel-to-pixel) correlated to chemical contrast (EsB detector), shown in image pairs A <=> B, C <=> D, and E <=> F. The low voltage mode of SEM allowed imaging of whole workflow stages from bare metal to completely functionalized sensor with bound bacteria, with no coating, due to low landing energies of the incident electron beam (800 eV). Real-time energy separation of back-scattered electrons allows chemical contrast to be generated; EsB detector grid in images (**B**, **D**, and **F**) set to—600 V to enrich single inelastic interaction-electrons characterized by low energy loss, LL-BSE, as described previously [22].

**Figure 8:**
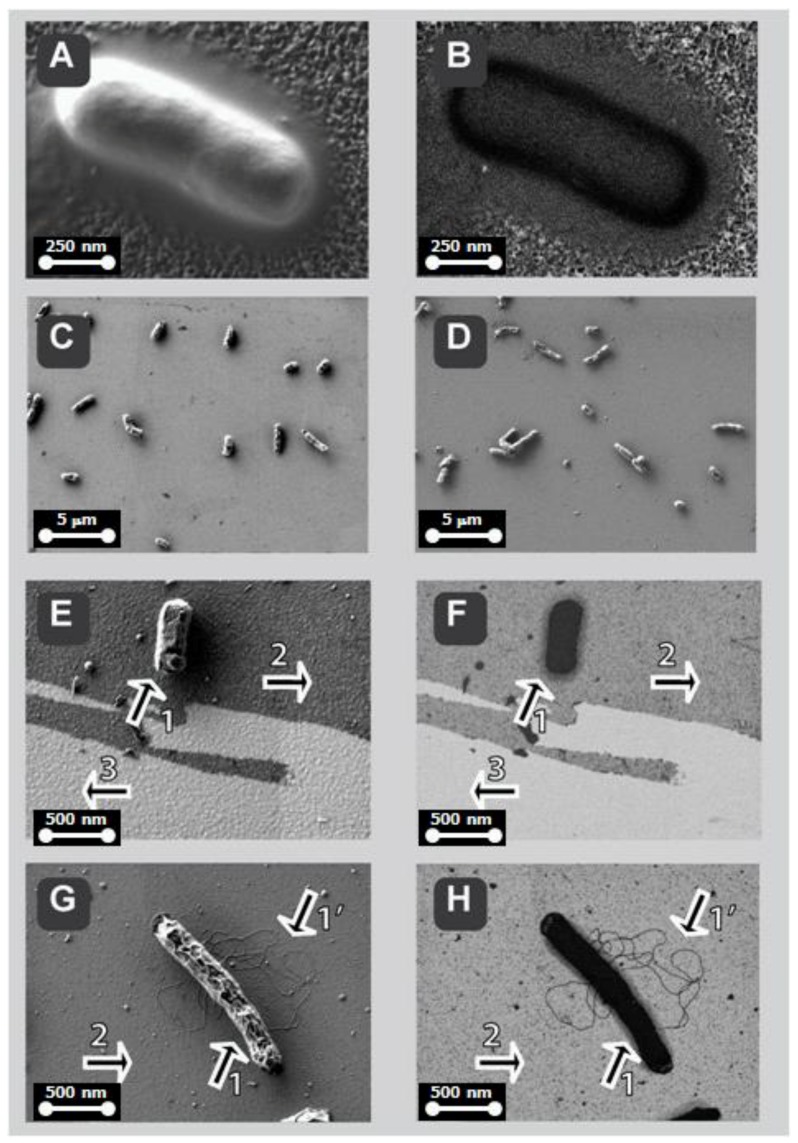
Functionalized biosensors for bacteria *Escherichia coli* of silicon chip metalized with titanium, gold, or ruthenium, visualized by scanning electron microscopy, SEM. The bacteria binding to functionalized Ti is shown in (**A**,**B**) as a result of specific interaction bacteria *Escherichia coli* and antibody anti-*Escherichia coli*. The surface of the functionalized sensor metallized with gold (**C**) versus ruthenium (**D**) shows the efficient binding of the bacterium *Escherichia coli*, at comparable efficacy for both metals. Enlarged images of bacteria bound to Au are shown in (**E**,**F**) or to Ru in (**G**, **H**). The topography/texture imaging was correlated to chemical contrast maps, shown in image pairs A <=> B, E <=> F, and G <=> H, with pixel-to-pixel accuracy. Advantages of correlative imaging clarify fine detail structures such as flagella of bacterium on functionalized ruthenium in Figure 8G <=> H (arrow 1’). Correlative imaging becomes particularly helpful when thin surface coatings (arrow 2) are to be distinguished from bare metallized-substratum (arrow 3), as shown in image pairs in Figure 8E <=> F and in Figure 7E <=> F.

Figure 7, illustrating low-voltage SEM imaging, shows the metallized-silicon (Figure 7A,B) and further functionalization steps, such as APTES-coating with glutaraldehyde fixation (Figure 7C,D) followed by antibody-immobilization on APTES (Figure 7E and Figure 7F, these two panels collectively show functionalized surface (arrow 2) and the underlying metallized substrate, delaminated by mechanical test scratching (arrow 3)). In Figure 7 panels A to F, the left panels show the topography/texture (A, C and E) while the right panels show the chemical contrast (B, D and F, correspondingly). Each pair of panels illustrates an exact pixel-to-pixel correlation, which was possible because of simultaneous recording in dual-channel mode (SE2-electrons with ET-detector for topography/texture while Low-Loss BSE electrons with EsB-detector, as described in detail previously [21,22]). *E*. *coli* bacteria and their filamentous extensions (Figure 7 and Figure 8, arrow 1 and 1’, dark appearance) become visible at high detail due to their high contrast against the metalized silicon substratum covered with thin layer of organic coat (arrow 2, grey appearance). In order to further explore the advantages of the correlative imaging, the biosensor was scratched to delaminate the functionalized coat, thus the underlying metallized surface became visible (arrow 3, bright appearance). In Figure 8, in a similar mode described for Figure 7, the topography/texture imaging was correlated to chemical contrast, shown in image pairs A <=> B, E <=> F and G <=> H, with pixel-to-pixel accuracy. These correlations demonstrate the clear advantage over single imaging, particularly obvious in fine detail structures such as flagella of bacterium in Figure 8 G <=> H (arrow 1’); the topography/texture correlation with chemical contrast appears a powerful tool also when thin surface coatings are to be distinguished from bare metallized-substratum, as shown by arrow 2 versus arrow 3 in in Figure 7 E <=> F and in Figure 8 E <=> F and G <=> H.

## 4. Discussion

Label-free sensors for bacteria detection are among the most promising tools in the field of bacteria detection. The development of highly sensitive, cost-effective, selective sensors requires the choice of a suitable sensor material, active molecules, and effective functionalization. Within this study, we showed how the sensor material may affect the ability of bacteria detection by biomolecules that demonstrated high selectivity and specificity of *Escherichia coli* detection in ELISA test. The present study is the first step for achieving effective sensor before the transducer strategies can be developed in form of microwave-based sensors. In microwave (electrical, in general) biosensors the metallic layers covering the substrate (typically silicone) are used for designing the sensor’s electrodes that constitute electrical circuit (Figure 2a). In a simple case it can take a form of an interdigital planar capacitor and the detection is based on capacitance measurements, when bacteria binding to the biofunctionalized sensor’s surface takes place [20]. Other types of biosensors can be found in literature among which one group can be classified as a group of resonant sensors with their frequency response being significant in a specified narrow frequency range [23,24,25] and a group of broadband sensors typically composed of transmission-line sections which exhibit response in a wide frequency range [26,27,28]. 

We studied several metals for composing conductor electrodes, for which Ti, Au, and Ru with very good electrical properties have been selected; the selected metals can be processed using well-established deposition methods on sensor surfaces, such as magnetron sputtering. In our research the metallic layers were deposited from metallic targets of Ti, Au, and Ru.

Titanium exhibits very good properties for biomedical applications, such as excellent biocompatibility and electrical conductivity. It has been mostly used for manufacturing metal orthopedic implant parts [29,30,31]. However, it can be also applied as an electrode material. Tang et al. [32] have presented the coated Ti anodes prepared for producing acidic electrolyzed oxidizing water (AEOW) [32], Nemati et al. [33] investigated magnetron-sputtered Ti-based thin films and Ti-based alloys for biomedical applications, the biocorrosion and biocompatibility was studied [33], Varshney and Li [34] have presented the results of bacterial cells detection by utilization of interdigitated array microelectrodes made of various materials, such as: Au, Pt, Ti, Cr, C, and ITO (indium tin oxide) [34]. 

Gold as an inert metal is not affected by oxidation and it is easy to manipulate using different fabrication techniques. Moreover, gold is compatible with cell culture, and SAMs bound on gold surfaces are stable for long periods after contact with biological media [35]. Furthermore, the well-known strong binding between thiolated molecules or biomolecules to gold surface offers multiple possibilities for surface functionalization [36], however care should be taken when antibodies are directly absorbed on the gold surface, because the denaturation and the reduction of their bioaffinity may occur [37].

Ruthenium is a transition metal belonging to the platinum group of the periodic table and exhibits unique advantages in comparison with other metals, including thermal stability, excellent corrosion resistance, low hysteresis, high sensitivity, and low resistivity. Due to the cost-efficient, highly sensitive, selective, and stable sensing properties ruthenium materials play a key role in developing NH_3_ sensors [38]. It has been also commonly used as a metal for electrodes applied to various applications, such as: electrocatalyst for the CO_2_ electroreduction [39], photoelectrocatalytic material [40], a porous anode for a flow-through direct methanol microfluidic fuel cell [41], or electrode for supercapacitors [42]. There are several methods to deposit Ru on the sensor surface, however, the two that are commonly applied for electrode deposition are magnetron sputtering [43,44,45] or hydrothermal [46,47,48]. Ruthenium in electrodes occurs as a single metal or in composition of two or more compounds depending on the application. It has been also successfully used in biomedical application, e.g., Afsharan et al. [49] have showed the highly sensitive electrochemiluminescence detection of p53 protein using functionalized Ru-silica nanoporous at gold nanocomposite, Guo et al. [50] have presented the ECL immunosensor fabricated of Ru layers to detect CA125 (Carbohydrate Antigen 125) and SCCA (Squamous Cell Carcinoma Antigen), whereas Zhang et al. [51] have used Ru(bpy)_3_^2+^-doped silica nanoparticles (Ru@SiO_2_ NPs) for highly sensitive detection of fumonisin B1 (FB1). 

The three different metals, Ti, Au, and Ru, were tested during this study. For specific biomolecules immobilization the metal surfaces were covered with stable silane layer in a controlled environment using APTES or GPTMS. The modifications of metal surfaces with self-assembled monolayers containing different molecules, such as thiol-terminated, amine-terminated, alkyl-terminated, and phenyl-terminated silanes, which allow attachment to various substrates through their terminal groups, have become increasingly popular [35]. The aminopropyltriethoxysilane has been the most extensively studied and probably is the most commonly known coupling agent used for surface functionalization [52,53,54,55,56,57,58]. APTES has three hydrolysable ethoxy groups that attach to the metal surface and the amine (-NH_2_) from the aminopropyl groups for further functionalization. It has been reported that the orientation and attachment of APTES on surfaces depend on temperature, humidity, concentration of APTES, and deposition time [59]. Gunda et al. [56] successfully coated silicon substrates by antibodies such as anti-human IgG, anti-myoglobin, or anti-dengue using APTES. Likewise, Gunda et al. [56] also implemented APTES on different metal surfaces to bind polyclonal anti-*E*. *coli* antibody. The metal surface functionalization by recombinant protein was performed by three-step procedure: silanization of the surface with an epoxy group suitable for reaction with NTA motif exhibiting primary amine, followed by Ni^2+^ loading. The GPTMS, which contains terminal epoxy groups, was used to create the epoxy-covered surface on metals. A similar procedure was used by Liu et al. [60], who showed that SiNWs can be chemically functionalized with Ni:NTA motifs using GPTMS, suitable for the specific immobilization of proteins via a short polyhistidine tag (His-tag) at close proximity to the SiNW surface. 

We noticed that for proteins which were covalently immobilized to the surface by glutaraldehyde (polyclonal antibody) the most effective was Ti, whereas for proteins, which were immobilized to the surface by His-tag-nickel reaction, the most effective was Ru. The observed differences may be related to the properties of metals. So, the surfaces in biosensors will be chosen depending on application. Both titanium and ruthenium are less demanding in magnetron-sputtering deposition, since they do not require an additional adhesive layer as it is necessary for gold deposition. It is worth considering that magnetron-sputtering deposition is fully compatible with CMOS (complementary metal-oxide-semiconductor) technology, which enables fabrication of fully integrated biosensors, such as Lab-on-Chip structures.

## 5. Conclusions

The three various metals: titanium (Ti), gold (Au), and ruthenium (Ru) have been used as potential materials for electrodes in microwave-based biosensors. The sensor surfaces were functionalized in two different strategies, moreover three different biological elements which are known to selectively recognize the *Escherichia coli* bacteria were used. Our investigations have shown, that for the detection strategy using polyclonal antibodies bound to the metal surface, the most effective was Ti. However, for a detection strategy using recombinant proteins tagged with His-Tag, the most efficient was Ru. 

Based on these results, our further investigation will be focused on electrical biosensor development in which the composition of Ti + APTES/glutaraldehyde + polyclonal antibodies will be used because of their good binding and selectivity properties. The sensor networks, which can take a form of e.g., a section of coupled lines where an increased sensitivity can be achieved when such a section is excited differentially, as described previously [61]. Furthermore, we aim at towards the development of a fully integrated microwave biosensor, where a sensing element with the necessary circuits will be integrated within a single chip.
